# Integration of Transcriptomic and Proteomic Approaches Reveals the Temperature-Dependent Virulence of *Pseudomonas plecoglossicida*

**DOI:** 10.3389/fcimb.2018.00207

**Published:** 2018-06-21

**Authors:** Lixing Huang, Wenjia Liu, Qingling Jiang, Yanfei Zuo, Yongquan Su, Lingmin Zhao, Yingxue Qin, Qingpi Yan

**Affiliations:** ^1^Fisheries College, Key Laboratory of Healthy Mariculture for the East China Sea, Ministry of Agriculture, Jimei University, Xiamen, China; ^2^State Key Laboratory of Large Yellow Croaker Breeding, Ningde, China; ^3^College of Ocean and Earth Sciences, Xiamen University, Xiamen, China

**Keywords:** white nodules disease, *Pseudomonas plecoglossicida*, virulence, temperature, transcriptome, proteomics

## Abstract

*Pseudomonas plecoglossicida* is a facultative pathogen that is associated with diseases of multiple fish, mainly at 15–20°C. Although fish disease caused by *P. plecoglossicida* has led to significant economic losses, the mechanisms of the temperature-dependent virulence are unclear. Here, we identify potential pathogenicity mechanisms and demonstrate the direct regulation of several virulence factors by temperature with transcriptomic and proteomic analyses, quantitative real-time PCR (qRT-PCR), RNAi, pyoverdine (PVD) quantification, the chrome azurol S (CAS) assay, growth curve measurements, a biofilm assay, and artificial infection. The principal component analysis, the heat map generation and hierarchical clustering, together with the functional annotations of the differentially expressed genes (DEGs) demonstrated that, under different growth temperatures, the animation and focus of *P. plecoglossicida* are quite different, which may be the key to pathogenicity. Genes involved in PVD synthesis and in the type VI secretion system (T6SS) are specifically upregulated at the virulent temperature of 18°C. Silencing of the PVD-synthesis-related genes reduces the iron acquisition, growth, biofilm formation, distribution in host organs and virulence of the bacteria. Silencing of the T6SS genes also leads to the reduction of biofilm formation, distribution in host organs and virulence. These findings reveal that temperature regulates multiple virulence mechanisms in *P. plecoglossicida*, especially through iron acquisition and T6SS secretion. Meanwhile, integration of transcriptomic and proteomic data provide us with a new perspective into the pathogenesis of *P. plecoglossicida*, which would not have been easy to catch at either the protein or mRNA differential analyses alone, thus illustrating the power of multi-omics analyses in microbiology.

## Introduction

The correlation between temperature and bacterial disease is of escalating concern because the virulence of an increasing number of pathogens is found to be regulated by temperature (Igbinosa and Okoh, 2008; Vezzulli et al., 2010; Hashizume et al., 2011; Lam et al., 2014). For example, *Pseudomonas syringae* pv. phaseolicola phytotoxin is optimally synthesized between 18 and 20°C, while no detectable amounts are present above 28°C (Aguilera et al., 2017).

Temperature regulation can be achieved at DNA, RNA, or protein level, and although several virulence factors could respond to temperature, the exact mechanisms of regulation still need to be investigated in many instances (Lam et al., 2014). Understanding how temperature regulates virulence factors is a significant challenge due to the complex regulatory networks governing gene expression and protein production in bacteria (Chen et al., 2013). Thus, omics analyses, for instance, genomics, transcriptomics, and proteomics, are emerging in the investigation of mechanisms involved in temperature-related infections. Currently, omics analyses are still performed individually with distinct approaches generating monothematic, rather than integrated knowledge (Manzoni et al., 2016). In fact, the genome, transcriptome, and proteome are not isolated biological entities, and multi-omics data should be concomitantly used and integrated to map mechanisms of temperature-related infections (Manzoni et al., 2016). Integration of transcriptomic and proteomic approaches could provide the multidimensional collection of a core set of genes that probably involved in temperature-regulated bacterial virulence (Miao et al., 2015).

*Pseudomonas plecoglossicida*, first isolated from ayu (*Plecoglossus altivelis*) (Zhang et al., 2014), is the cause of bacterial hemorrhagic ascites in freshwater fish. To date, infection of ayu, large yellow croaker (*Pseudosciaena crocea*), pejerrey (*Odontesthes bonariensis*), and rainbow trout (*Oncorhynchus mykiss*) with *P. plecoglossicida* has been reported (Akayli et al., 2011; Mao et al., 2013; Hu et al., 2014; Zhang et al., 2014). Outbreaks of *P. plecoglossicida* infection in cage-farmed *P. crocea*, which is characterized by white nodules in the internal organs of infected fish (including kidney, spleen, and liver) and causes high mortality, have led to severe economic losses in the Fujian and Zhejiang provinces of China (Zhang et al., 2014). Meanwhile, our group performed artificial infection of orange spotted grouper (*Epinephelus coioides*) with *P. plecoglossicida* and observed the same symptoms, i.e., white nodules in the internal organs. Therefore, *P. plecoglossicida* might be a potent pathogen of other species of fish and should receive more attention.

Previous studies were mainly focused on the identification of the etiological agent of white nodules in *P. crocea*. Further investigation of the mechanism underlying the pathogenesis of *P. plecoglossicida* could provide potential anti-*P. plecoglossicida* targets. Mao et al. (2013) reported the draft genome sequence of *P. plecoglossicida* strain NB2011, which was isolated in Ningbo from diseased *P. crocea* with white nodules. In our previous study, a strain of *P. plecoglossicida* (NZBD9) isolated in Ningde in April 2013 from the internal organs of a diseased *P. crocea* with white nodules was used to perform the genome sequencing, too (Huang et al., 2018). Both genome sequences reveal several virulence factors including type IV pilus, multiple flagellins, type III secretion system (T3SS), the iron uptake system, and type VI secretion system (T6SS) (Mao et al., 2013; Huang et al., 2018). However, further research is needed to investigate whether and how these systems work during the pathogenesis of *P. plecoglossicida*.

Since the disease outbreaks often occur between April and May, we hypothesized that the virulence of *P. plecoglossicida* is temperature-dependent. To verify this hypothesis, NZBD9 was used to perform the artificial infection of large yellow croaker and *Epinephelus coioides* at different temperatures. Artificial infection with NZBD9 caused disease in the internal organs of large yellow croaker and *E. coioides* at 16–19°C but not at 7–12 and 24–28°C. The results indicated that the virulence of *P. plecoglossicida* is temperature-dependent, although little is known regarding the specific mechanisms involved in temperature-related *P. plecoglossicida* infection.

In the present study, in order to investigate the mechanisms involved in the temperature-related *P. plecoglossicida* infection, we compared the influence of non-pathogenic (12 and 28°C) and pathogenic (18°C) temperatures on virulence factors expression in NZBD9 with RNA-seq and iTRAQ.

## Materials and methods

### Bacterial strain and culture conditions

Pathogenic *P. plecoglossicida* strain NZBD9 was isolated from naturally infected large yellow croaker and identified by time-of-flight mass spectrometry (Hu et al., 2014), 16S rRNA sequencing and artificial infection. *P. plecoglossicida* was cultured overnight with shaking (220 r.p.m.) in Luria–Bertani (LB) broth at 12, 18, and 28°C.

Bacterial suspensions were adjusted to OD_560_ = 0.3 for genome/RNA/protein extraction as well as other experiments.

*Escherichia coli* SM10 (Dongsheng, Guangzhou, China) was grown at 37°C in LB broth (220 r.p.m.) or on LB agar plates.

### RNA-seq

Total RNA were extracted from NZBD9 cultured at 12, 18, and 28°C. Three replicates were prepared for each treatment. Sequencing was performed at CapitalBio Technology (Beijing, China) with Ion Proton 200 Sequencing Kit v3 (Life Technologies) on the P1 ion chip. Data were collected with Torrent Suite v4.0 software.

Clean reads were mapped to the reference genome sequence of NZBD9 (accession number: PHNR00000000) using SOAP2 (Li et al., 2009). Mismatches (≤ 5 bases) were allowed during the alignment. The unigene expression was calculated by the reads per kb per million reads (RPKM) method (Mortazavi et al., 2008). Two classes unpaired MA-plot-based method was applied to detect and visualize gene expression differences with significant *P*-value < 0.001 (differential ratio ≥2) between samples cultured at different temperatures.

Blast2GO program was used to obtain GO annotations of the unigenes. After the GO annotation for every gene was acquired, WEGO software was used to carry out GO functional classification for all genes and understand the gene functions distribution at the macro level. *P*-value was then subjected to Bonferroni Correction with corrected *P-*value ≤ 0.05 as a threshold. Significantly enriched GO terms in DEGs were identified by GO terms fulfilling this condition.

The COG and KEGG pathway annotations were performed with Blastall software against the COG (http://www.ncbi.nlm.nih.gov/COG) as well as KEGG (http://www.genome.jp/kegg/) databases. Pathways with *Q*-values ≤ 0.05 were regarded as significantly enriched in DEGs.

### Proteome analysis

The extracellular and intracellular proteins were separately extracted from NZBD9 cultured at 12, 18, and 28°C (*n* = 3). The proteins were then used for proteome analysis. Labeling of the samples with iTRAQ reagents was carried out as previously described by Ye et al. (2010). The fractionation was carried out as previously described by Zeng et al. (2013). LC-MS/MS analyses were carried out with an LTQ Orbitrap Velos (Thermo Fisher Scientific) with an Agilent 1,100 binary high-pressure liquid chromatography (HPLC) pump (Agilent Technologies) and a Famos autosampler (LC Packings). Scaffold Q+ was used for protein identification and quantification with iTRAQ (Glen et al., 2008). Further quantitative data analyses were qualified on unique proteins with at least two unique peptides and a false discovery rate (FDR) < 0.01. The fold changes in protein abundance were defined as the median ratios of all significantly matched spectra with tag signals. Proteins expressed differentially between samples cultured at different temperatures were identified as fold changes ≥1.5 (*P*-value < 0.001).

### Stable gene silencing

Stable gene silencing was carried out according to the description of Wang et al. (2015). Short hairpin RNA sequences targeting the coding regions of mRNAs were obtained from Shanghai Generay Biotech Co., Ltd. (Shanghai, China) (Table S1). The annealed oligonucleotides were ligated to the *Bam*HI and *Sph*I (TaKaRa, Japan) double-digested pACYC184 vector with T4 DNA ligase (TaKaRa, Japan). Recombinant plasmids were then transformed into *P. plecoglossicida* by electroporation. An empty pACYC184 vector was used as a control. Stable silenced clones were screened by chloramphenicol (34 μg/ml).

### Quantitative real-time PCR (qRT-PCR)

TRAzol (Dongsheng, Guangzhou, China) was used for Total RNA extraction as described by Huang et al. (2015). Revert Aid Mu-MLV cDNA synthesis kit (Dongsheng, Guangzhou, China) was used to synthesize first-strand cDNA from the total RNA.

qRT-PCR analysis was carried out on a QuantStudio™ 6 Flex real-time PCR system (ABI, USA) using SYBR green I fluorescent dye (Dongsheng, Guangzhou, China) following the manufacturer's instructions. The primers were designed with Primer Premier 5.0 and listed in Table S2. The expression levels were normalized using 16S RNA, and mean expression levels were calculated using the 2^−ΔΔ*Ct*^ method (*n* = 6). The primers are listed in Table S2.

To detect the dynamic distribution of *P. plecoglossicida* in host, the copy number of the *gyrB* gene was used to estimate *P. plecoglossicida* abundance in spleens, trunk kidneys, head kidneys, blood, and livers. The primers are listed in Table S2. The *gyrB* DNA copy number per milligram of tissue was used to express the result.

### Artificial infection

For survival assays, one-hundred-and-forty healthy individuals of *E. coioides* were divided into 7 groups randomly. Each fish was injected intraperitoneally with 0.1 mL of wild-type and stable silenced strains of *P. plecoglossicida* (1 × 10^7^ cfu/ml) at 18°C (Liu et al., 2017). Instead of the *P. plecoglossicida* suspension, sterile PBS was used as a negative control. Infected fish were observed for morbidity and mortality daily for 15 days.

For the tissue distribution assays, weight-matched *E. coioides* were infected with *P. plecoglossicida*. The spleens, trunk kidneys, head kidneys, blood and livers of three *E. coioides* were sampled at 1, 6, 12, 24, 48, 72, and 96 h after being injected with the wild-type *P. plecoglossicida* or RNAi strains, respectively.

### DNA isolation

DNA purification from spleens, trunk kidneys, head kidneys, blood and livers was accomplished with an EasyPure Marine Animal Genomic DNA Kit (TransGen Biotech, Beijing, China) according to the manufacturer's instructions. The EasyPure Blood Genomic DNA Kit (TransGen Biotech) was used for DNA isolation from blood.

### PVD quantification

Infinite 500 Plate Reader (Tecan, Crailsheim) was used to quantify PVD in the supernatants of the cultures by measuring fluorescence emission at 485 nm after excitation at 420 nm.

### Chrome azurol S (CAS) assay

CAS-agar plates were prepared through mixing LB growth media with a dye made of Fe, CAS and hexadecyl-trimethyl-ammonium bromide (HDTMA) (Tanui et al., 2017). Sterilized filter paper discs with a diameter of 10 mm were placed on agar plates overnight. A 10 μL supernatant of bacteria was diffused onto the paper disc. The uninoculated plates of CAS-agar were used as control. Three replicates for each repetition were carried out. The distance of the advancing color-change front in the CAS-blue agar from the center of the paper-disc was measured to determine the CAS reaction.

### Growth curves in the presence of 2,2′-dipyridyl

The wild-type and gene silenced *P. plecoglossicida* were grown at 28°C in LB overnight and then the OD_600nm_ of the cultures were adjusted to 0.2. The wild-type and gene silenced *P. plecoglossicida* were then incubated at 18°C in the presence or absence of 100 μM 2,2′-dipyridyl (Sigma) (*n* = 3). The values of OD_600nm_ were measured at 1, 4, 7, 10, 13, 16, 19, 22, 25, and 28 h after the addition of 2,2′-dipyridyl. From the OD_600nm_ data, growth curves were plotted to compare the wild-type and silenced strains in the presence and absence of 2,2′-dipyridyl.

### Biofilm assay

The biofilm assay for *P. plecoglossicida* was carried out as described by Luo et al. (2016). *P. plecoglossicida* strains were grown at 28°C in LB overnight and then the OD_600nm_ of the cultures were adjusted to 0.2.

150 μl of LB was mixed with 50 μl of bacterial culture, and then incubated at 18°C. After incubation for 24 h, it was washed using sterile PBS for 3 times, stained for 15 min with 200 μl 1% crystal violet, rinsed again with sterile PBS, and then air dried. Two hundred microliter acetic acid (33%) was used for solubilizing the stained biofilm, which was quantitated through measuring OD_590nm_. Six replicates were performed for each treatment.

### Data processing

Results are reported as means ± S.E. Statistical analysis was conducted with SPSS 13.0 software (SPSS, Chicago, IL, USA). Differences were analyzed with one-way analysis of variance (ANOVA) followed by Dunnett's multiple comparison test. A value of *P* < 0.05 was considered as a significant difference.

### Ethics statement

All animal experiments were carried out strictly under the recommendations in the “Guide for the Care and Use of Laboratory Animals” set by the National Institutes of Health. The animal protocols were approved by the Animal Ethics Committee of Jimei University (Acceptance NO JMULAC201159).

## Results

### NZBD9 transcriptome

**Figure 1:**
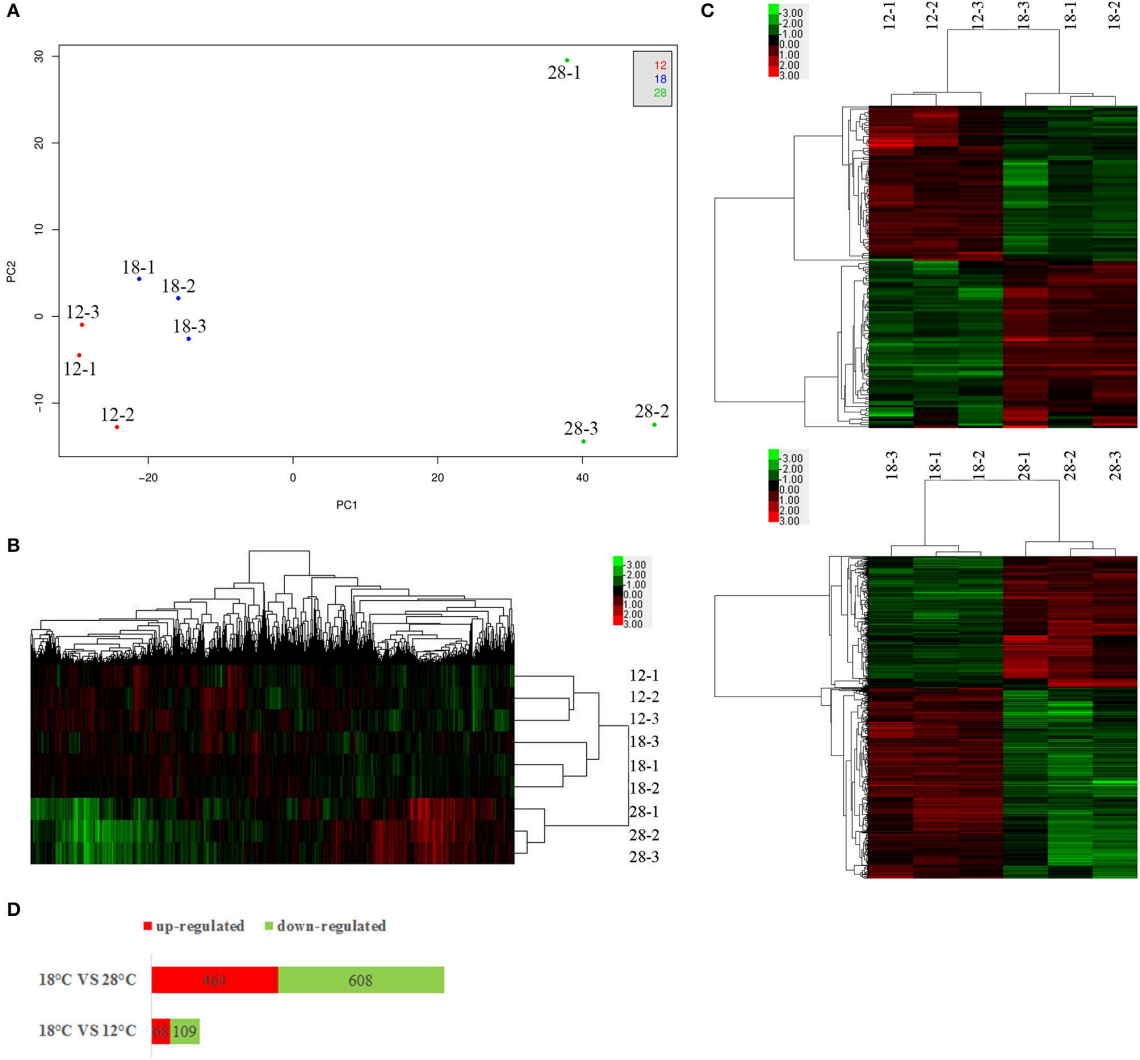
PCA analysis **(A)**, hierarchical clustering of all genes **(B)** and the DEGs **(C)**, and numbers of DEGs **(D)**. For each temperature treatment, there are three replicates (12-1, 12-2, 12-3; 18-1, 18-2, 18-3; 28-1, 28-2, 28-3). The plot demonstrates clear separation of the 3 sample groups **(A)**. For hierarchical clustering, green and red indicate decreased and increased expression, respectively. Transcripts were clustered by hierarchical clustering using the complete linkage algorithm and Pearson correlation metric in R.

To analyze the expression of genes in NZBD9, we used RNA-seq to compare genes expressed at the avirulent (12 and 28°C) and virulent (18°C) temperatures of this strain and observed significant changes in gene expression (Figure 1). The result has been deposited in the NCBI sequence read archive (accession number SRP107111).

The principal component analysis (PCA) revealed a clear segregation of samples cultured at different temperatures (Figure 1A). The PCA plot also showed that samples cultured at different temperatures were generally clustered together. Meanwhile, the heat map generation and hierarchical clustering were performed on all samples and showed that the expression profiles of genes from samples cultured at different temperatures were clearly distinguishable (Figure 1B).

Compared to the samples cultured at 12°C, our results revealed significant regulation of 177 genes (68 upregulated and 109 downregulated) in NZBD9 grown at 18°C (Figures 1C,D). Compared to the samples cultured at 28°C, our results revealed significant regulation of 1,072 genes (464 upregulated and 608 downregulated) in NZBD9 grown at 18°C (Figures 1C,D).

**Figure 2:**
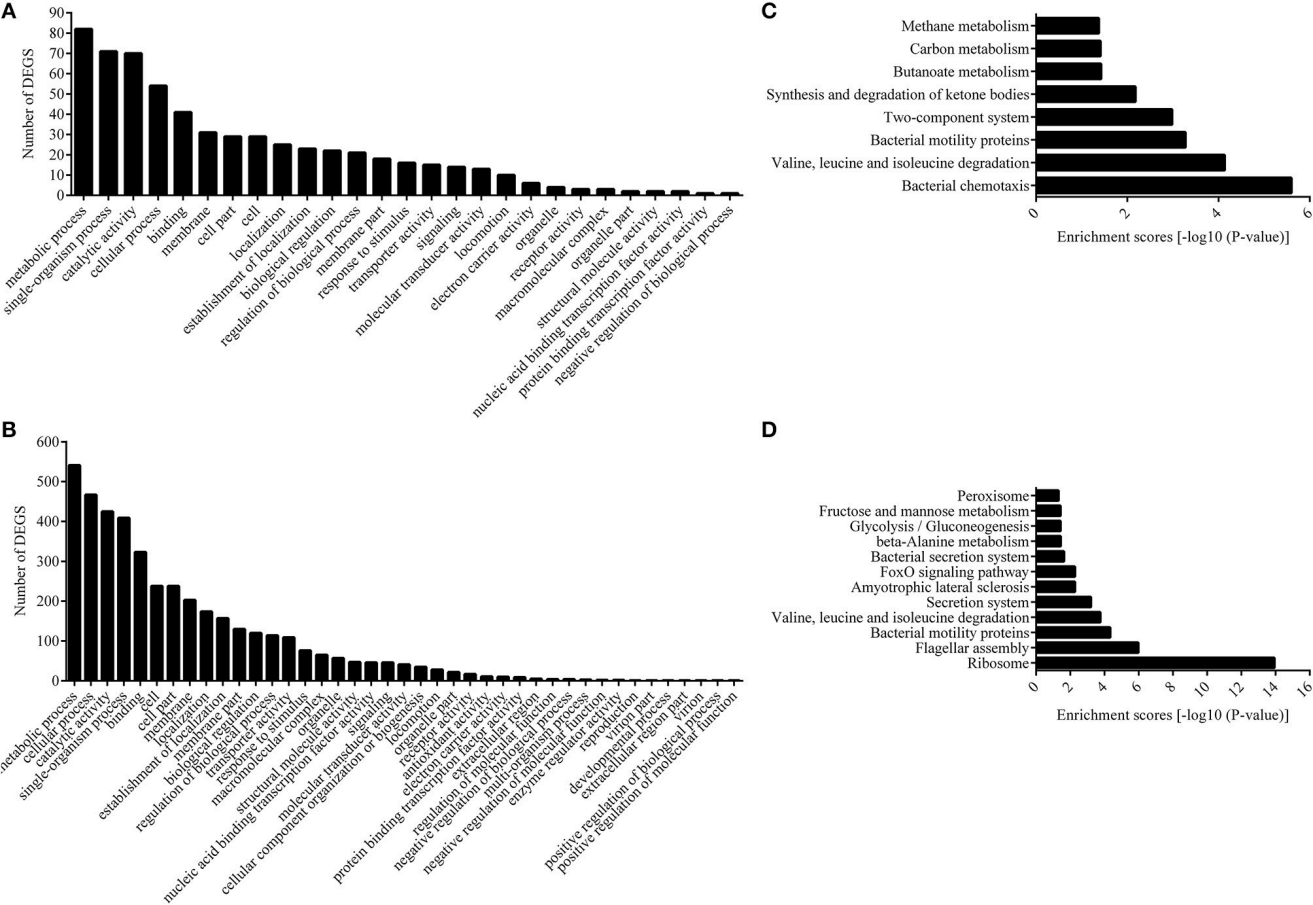
Functional annotation of DEGs based on GO and KEGG database. Histogram presentation of clusters of GO classification of DEGs in 12°C **(A)** and 28°C group **(B)**. Distributions of the KEGG pathways in 12°C **(C)**, and 28°C group **(D)**.

The functions of the differentially expressed genes (DEGs) were analyzed by GO, and the number of genes mapped to every term was counted. This analysis categorized 177 and 1,072 DEGs from the 12 and 28°C treated groups into 27 and 41 enriched functional groups, respectively (Figures 2A,B). GO categories analysis showed that, even though the number of enriched functional groups was not the same, the chief functional distribution of the DEGs from each group was similar. Most of the DEGs were involved in the following functional categories: metabolic process, single-organism process, catalytic activity, cellular process, binding, membrane, cell part, cell, and localization (Figures 2A,B).

We also assigned functions to all the DEGs according to COG, while the DEGs were annotated to 3,348 functions involved in 25 COG categories. Meanwhile, no DEG was annotated to “RNA processing and modification,” “chromatin structure and dynamics,” “nuclear structure,” “cytoskeleton,” and “extracellular structure” (Figures S1, S2). The distributions of the DEGs from the 12°C- and 28°C-treated groups were quite different. DEGs from the 12°C-treated groups were mainly annotated to “signal transduction mechanisms,” “amino acid transport and metabolism,” and “lipid transport and metabolism,” while DEGs from 28°C-treated groups were mainly annotated to “translation, ribosomal structure, and biogenesis,” “transcription,” and “energy production and conversion.”

Next, we carried out pathway analysis of DEGs on the basis of the latest version of the KEGG database. Top-ranking biological pathways with significant enrichment of DEGs were identified (*p*-value cutoff 0.05) (Figures 2C,D). Again, the top-ranking biological pathways were different between samples from the 12- and 28°C-treated groups.

**Figure 3:**
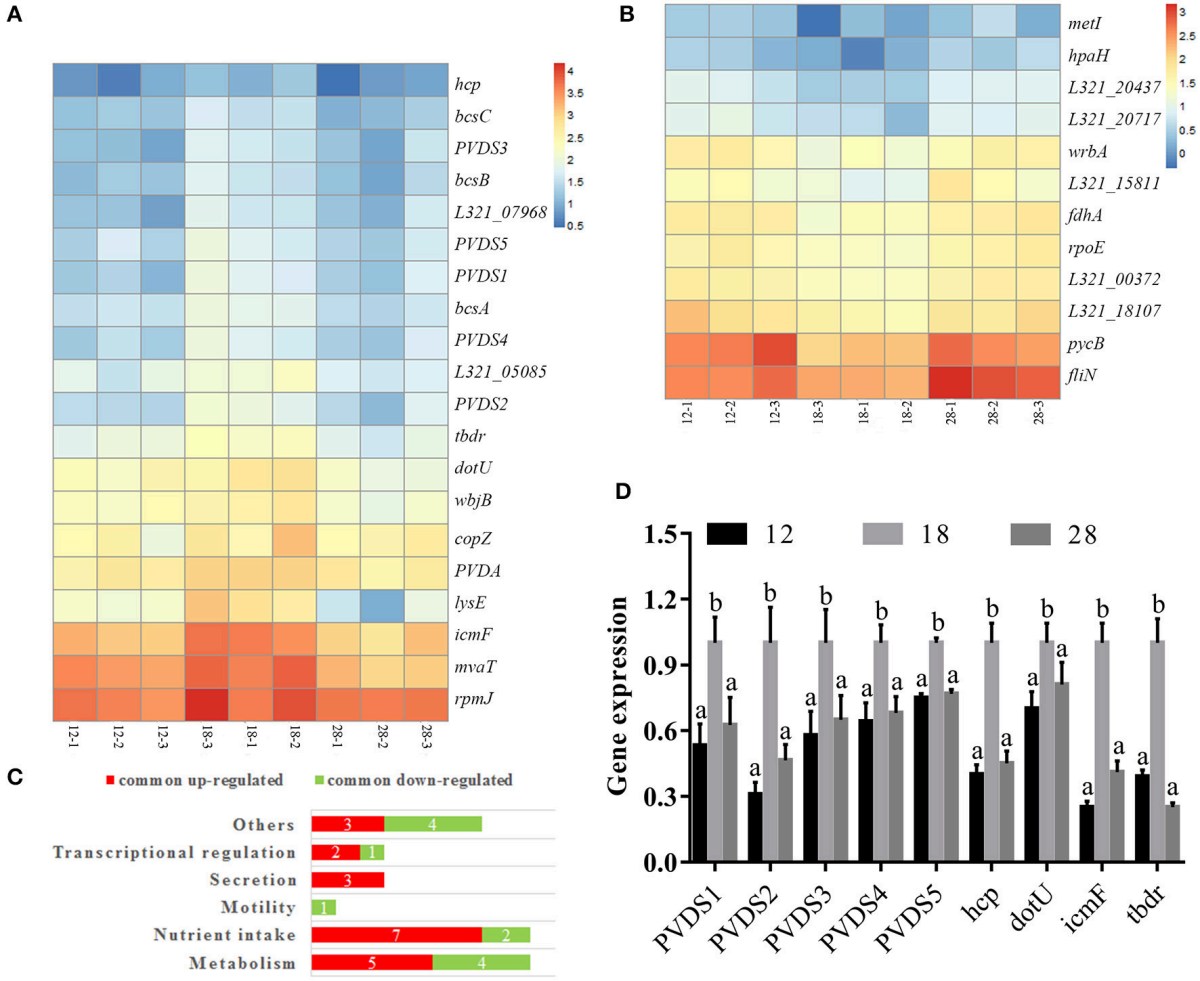
Hierarchical clustering of commonly up-regulated **(A)** and down-regulated DEGs **(B)**, numbers of DEGs in enriched functions **(C)**, and the validation of RNA-seq by qRT-PCR **(D)**. For hierarchical clustering **(A)**, blue and red indicate decreased and increased expression, respectively. Transcripts were clustered by hierarchical clustering using the complete linkage algorithm and Pearson correlation metric in R. For QPCR analysis of the expression of randomly selected novel genes **(B)**, data are presented as mean ± S.D. (*n* = 3). Means of treatments not sharing a common letter are significantly different at *P* < 0.05.

The common DEGs from the 12°C- and 28°C-treated groups were identified (Table S3); 20 DEGs were specifically upregulated at 18°C (Figure 3A), and 12 DEGs were specifically downregulated at 18°C (Figure 3B). These DEGs included factors involved in metabolism, nutrient intake, motility, secretion and transcriptional regulation (Figure 3C). Among the 20 DEGs specifically upregulated at 18°C, 2 were identified as pyoverdine (PVD) synthetases and were named PVDS1 and PVDS2, and 3 DEGs were identified as PVD sidechain peptide synthetases and were named PVDS3, PVDS4 and PVDS5. Meanwhile, 1 DEG was identified as a TonB-dependent siderophore receptor (TBDR). 3 DEGs were identified as *hcp, icmF*, and *dotU*. These genes belong to the type VI secretion system (T6SS). Compared to the samples cultured at 12°C, our results revealed significant up-regulation of PVDS1, PVDS2, PVDS3, PVDS4, PVDS5, *tbdr, hcp, icmF*, and *dotU* in NZBD9 grown at 18°C by 4.06-, 3.03-, 3.95-, 3.03-, 2.27-, 2.23-, 2.14-, 2.29-, and 3.03-fold, respectively (Table S3). Compared to the samples cultured at 28°C, our results revealed significant up-regulation of PVDS1, PVDS2, PVDS3, PVDS4, PVDS5, *tbdr, hcp, icmF*, and *dotU* in NZBD9 grown at 18°C by 2.35-, 3.21-, 2.22-, 2.44-, 2.38-, 3.04-, 2.39-, 3.96-, and 2.92-fold, respectively (Table S3).

To validate the results from the transcriptomic analysis, qRT-PCR and proteomic analysis were performed. Compared to the samples cultured at 12°C, our qRT-PCR results revealed significant up-regulation of PVDS1, PVDS2, PVDS3, PVDS4, PVDS5, *tbdr, hcp, icmF*, and *dotU* in NZBD9 grown at 18°C by 1.88-, 3.23-, 1.73-, 1.56-, 1.33-, 2.56-, 2.50-, 1.43-, and 4.00-fold, respectively (Figure 3D). Compared to the samples cultured at 28°C, our qRT-PCR results revealed significant up-regulation of PVDS1, PVDS2, PVDS3, PVDS4, PVDS5, *tbdr, hcp, icmF*, and *dotU* in NZBD9 grown at 18°C by 1.60-, 2.15-, 1.54-, 1.47-, 1.30-, 4.03-, 2.22-, 1.23-, and 2.44-fold, respectively (Figure 3D). The qRT-PCR results generally matched those of the sequencing, which reinforced the reliability of the RNA-seq.

### Integrated analysis of the transcriptome and proteome

iTRAQ was used to compare the expression of proteins at 12, 18, and 28°C. The result of iTRAQ has been deposited to the ProteomeXchange Consortium via the PRIDE partner repository with the dataset identifier PXD009586. Furthermore, the integration of the proteomic and transcriptomic data were analyzed.

**Figure 4:**
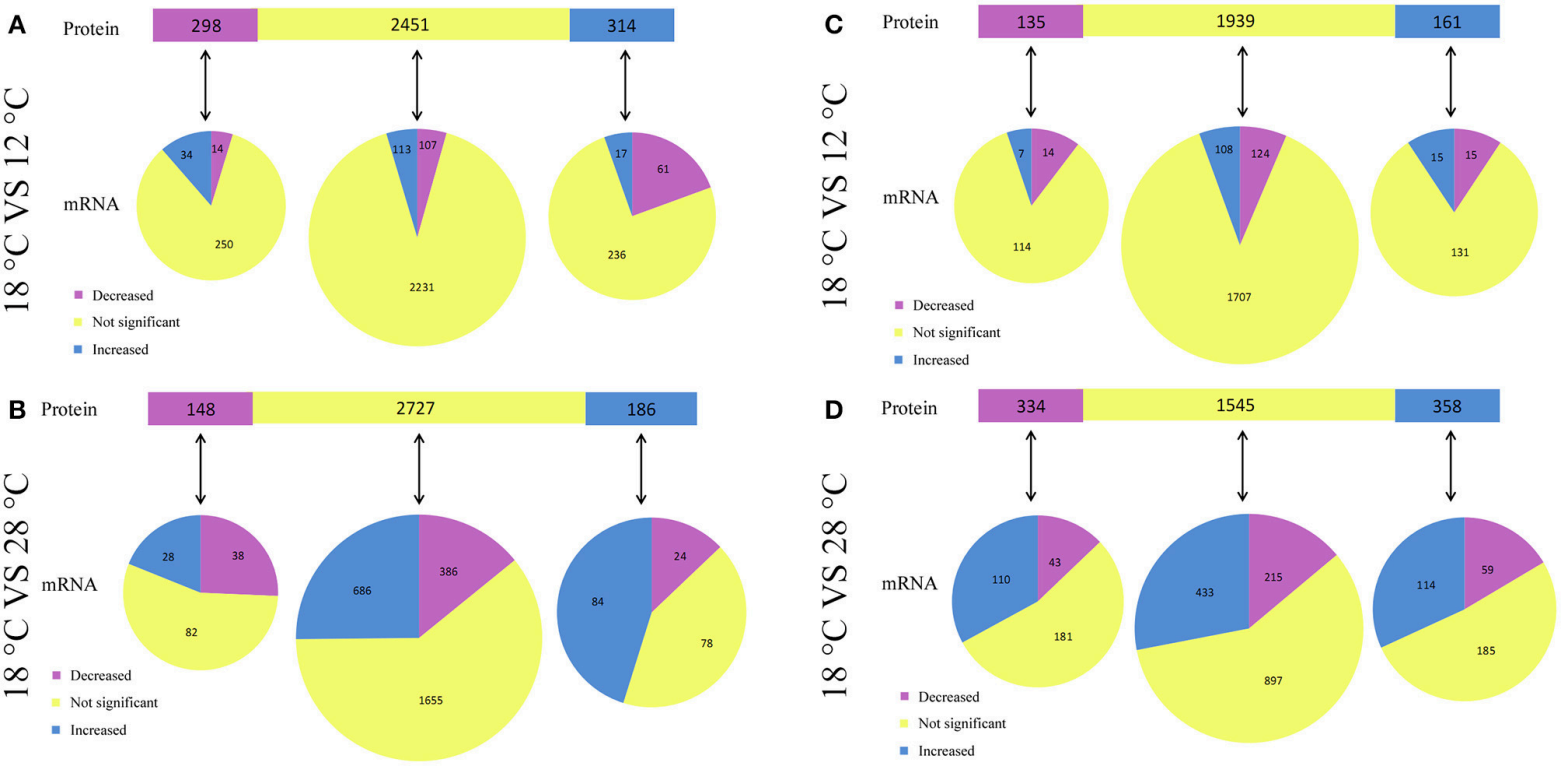
Relationship between protein changes and transcript changes in response to different temperatures. For summarization of proteins that have mRNA measurements, proteins (top bar) are grouped with regard to their responses to temperature. Each protein group was then subgrouped with regard to corresponding mRNA changes (bottom pies). The comparison between intracellular protein changes and transcript changes were listed in **(A,B)**, while the comparison between extracellular protein changes and transcript changes were listed in **(C,D)**.

Compared to the samples cultured at 12°C, our results revealed significant regulation of 612 (314 upregulated and 298 downregulated) intracellular proteins in NZBD9 grown at 18°C (Figure 4A). Among the 314 upregulated proteins, 17 of the corresponding encoding genes were also significantly upregulated, while 61 of the corresponding encoding genes were downregulated. Among the 298 downregulated proteins, 14 of the corresponding encoding genes were also significantly downregulated, while 34 of the corresponding encoding genes were upregulated. Therefore, there were 31 proteins displaying similar expression profiles as their corresponding encoding genes.

Compared to the samples cultured at 28°C, our results revealed significant regulation of 334 (186 upregulated and 148 downregulated) intracellular proteins in NZBD9 grown at 18°C (Figure 4B). Among the 186 upregulated proteins, 84 of the corresponding encoding genes were also significantly upregulated, while 24 of the corresponding encoding genes were downregulated. Among the 148 downregulated proteins, 38 of the corresponding encoding genes were also significantly downregulated, while 28 of the corresponding encoding genes were upregulated. Therefore, there were 122 proteins displaying similar expression profiles as their corresponding encoding genes.

Compared to the samples cultured at 12°C, our results revealed significant regulation of 296 (161 upregulated and 135 downregulated) extracellular proteins in NZBD9 grown at 18°C (Figure 4C). Among the 161 upregulated proteins, 15 of the corresponding encoding genes were also significantly upregulated, while 15 of the corresponding encoding genes were downregulated. Among the 135 downregulated proteins, 14 of the corresponding encoding genes were also significantly downregulated, while 7 of the corresponding encoding genes were upregulated. Therefore, there were 29 proteins displaying similar expression profiles as their corresponding encoding genes.

Compared to the samples cultured at 28°C, our results revealed significant regulation of 692 (358 upregulated and 334 downregulated) extracellular proteins in NZBD9 grown at 18°C (Figure 4D). Among the 358 upregulated proteins, 114 of the corresponding encoding genes were also significantly upregulated, while 59 of the corresponding encoding genes were downregulated. Among the 334 downregulated proteins, 43 of the corresponding encoding genes were also significantly downregulated, while 110 of the corresponding encoding genes were upregulated. Therefore, there were 157 proteins displaying similar expression profiles as their corresponding encoding genes.

Interestingly, Hcp was detected in the extracellular proteins and was found to be specifically upregulated at 18°C. Compared to the samples cultured at 12°C, our iTRAQ results revealed significant up-regulation of Hcp in the extracellular proteins of NZBD9 grown at 18°C by 4.81-fold (Table S3). Compared to the samples cultured at 28°C, our iTRAQ results revealed significant up-regulation of Hcp in the extracellular proteins of NZBD9 grown at 18°C by 6.41-fold (Table S3). Meanwhile, the encoding proteins of the 5 PVD-synthesis-related genes were detected in the cell and found to be specifically upregulated at 18°C. Compared to the samples cultured at 12°C, our iTRAQ results revealed significant up-regulation of PVDS1, PVDS2, PVDS3, PVDS4, and PVDS5 in the intracellular proteins of in NZBD9 grown at 18°C by 1.77-, 2.00-, 1.56-, 2.08-, and 2.13-fold, respectively (Table S3). Compared to the samples cultured at 28°C, our iTRAQ results revealed significant up-regulation of PVDS1, PVDS2, PVDS3, PVDS4, and PVDS5 in the intracellular proteins of in NZBD9 grown at 18°C by 2.54-, 1.93-, 1.51-, 2.15-, and 1.87-fold, respectively (Table S3). These results generally matched those of the RNA-seq and qRT-PCR, which reinforced the reliability of the iTRAQ.

### Temperature-dependent regulation of PVD synthesis is involved in the virulence of *P. plecoglossicida*

**Figure 5:**
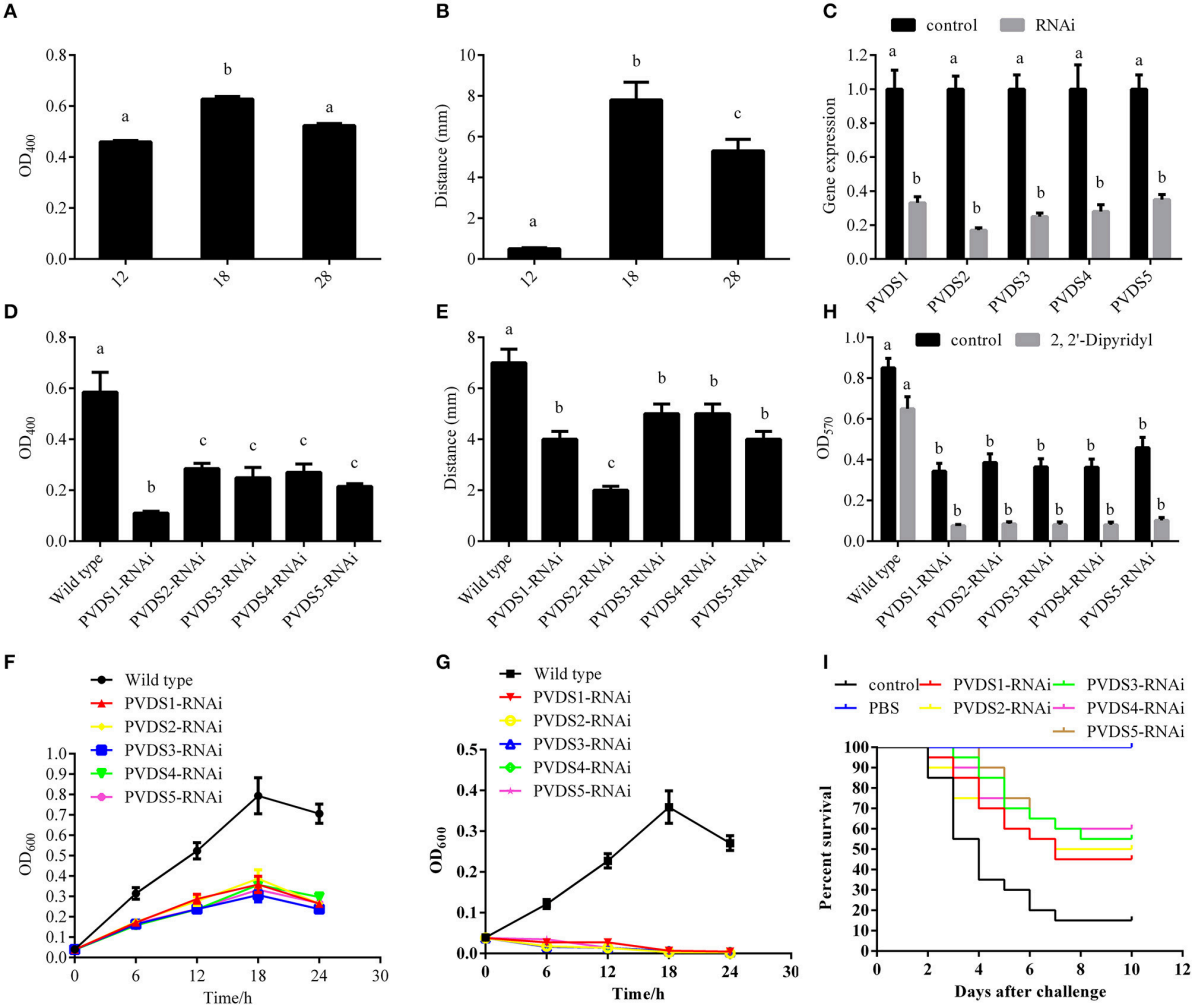
Temperature-dependent regulation of PVD synthesis involved in virulence of *Pseudomonas plecoglossicida*. **(A)** Presence of PVD in the supernatants of cultures under different temperatures. Data are presented as mean ± S.D. (*n* = 3). Means of treatments not sharing a common letter are significantly different at *P* < 0.05. **(B)** The distance of the advancing color-change front in the CAS-blue agar under different temperatures. Data are presented as mean ± S.D. (*n* = 3). Means of treatments not sharing a common letter are significantly different at *P* < 0.05. **(C)** qRT-PCR analysis of the expression of *pvds1, pvds2, pvds3, pvds4*, and *pvds5* after stable gene silencing compared with the control. Data are presented as mean ± S.D. (*n* = 3). Means of treatments not sharing a common letter are significantly different at *P* < 0.05. **(D)** Presence of PVD in the supernatants of stable silenced strains compared with the control. Data are presented as mean ± S.D. (*n* = 3). Means of treatments not sharing a common letter are significantly different at *P* < 0.05. **(E)** The distance of the advancing color-change front in the CAS-blue agar of stable silenced strains compared with the control. Data are presented as mean ± S.D. (*n* = 3). Means of treatments not sharing a common letter are significantly different at *P* < 0.05. **(F)** Growth of wild-type and stable silenced strains in the absence of 2, 2′-Dipyridyl (*n* = 3). **(G)** Growth of wild-type and stable silenced strains in the presence of 2, 2′-Dipyridyl (*n* = 3). **(H)** Biofilm formation of wild-type and stable silenced strains in the presence or absence of 2, 2′-Dipyridyl. Data are presented as mean ± S.D. (*n* = 6). Means of treatments not sharing a common letter are significantly different at *P* < 0.05. **(I)** The cumulative survival of *E. coioides* injected with wild-type and *pvds1*-, *pvds2*-, *pvds3*-, *pvds4*-, and *pvds5*-RNAi strains during 10 days post-challenge.

According to the integrated analysis of the transcriptome and proteome, we hypothesized a close connection between the 5 PVD-synthesis-related genes and the temperature-dependent virulence of *P. plecoglossicida*. To validate this hypothesis, the synthesis of PVD in the supernatants of cultures was quantified at avirulent and virulent temperatures (Figures 5A,B). Presence of PVD in the supernatants of cultures under 12, 18, and 28°C were 0.56 ± 0.005, 0.63 ± 0.010, and 0.58 ± 0.08, respectively (Figure 5A). The distance of the advancing color-change front in the CAS-blue agar under 12, 18, and 28°C were 0.50 ± 0.056, 7.80 ± 0.867, and 5.30 ± 0.589, respectively (Figure 5B). Taken together, our results showed that the synthesis of PVD was significantly elevated at 18°C, which was consistent with the mRNA and protein expression profile of the 5 PVD-synthesis-related genes. These 5 genes were then silenced with RNAi, which significantly reduced their expression by 3.03-, 5.88-, 4.00-, 3.57-, and 2.86-fold, respectively (Figure 5C). The synthesis of PVD at 18°C was significantly reduced in the silenced strains (Figures 5D,E). Presence of PVD in the supernatants of PVD-RNAi cultures were reduced by 5.31-, 2.05-, 2.35-, 2.17-, and 2.72-fold, respectively (Figure 5D). The distance of the advancing color-change front in the CAS-blue agar after PVD-RNAi were reduced by 2.87-, 2.25-, 1.29-, 1.29-, and 2.87-fold, respectively (Figure 5E). Therefore, we confirmed that the 5 genes were involved in PVD synthesis, and these genes were regulated by temperature. Meanwhile, growth, biofilm formation and virulence of NZBD9 were significantly decreased in the silenced strains (Figures 5F–I).

**Figure 6:**
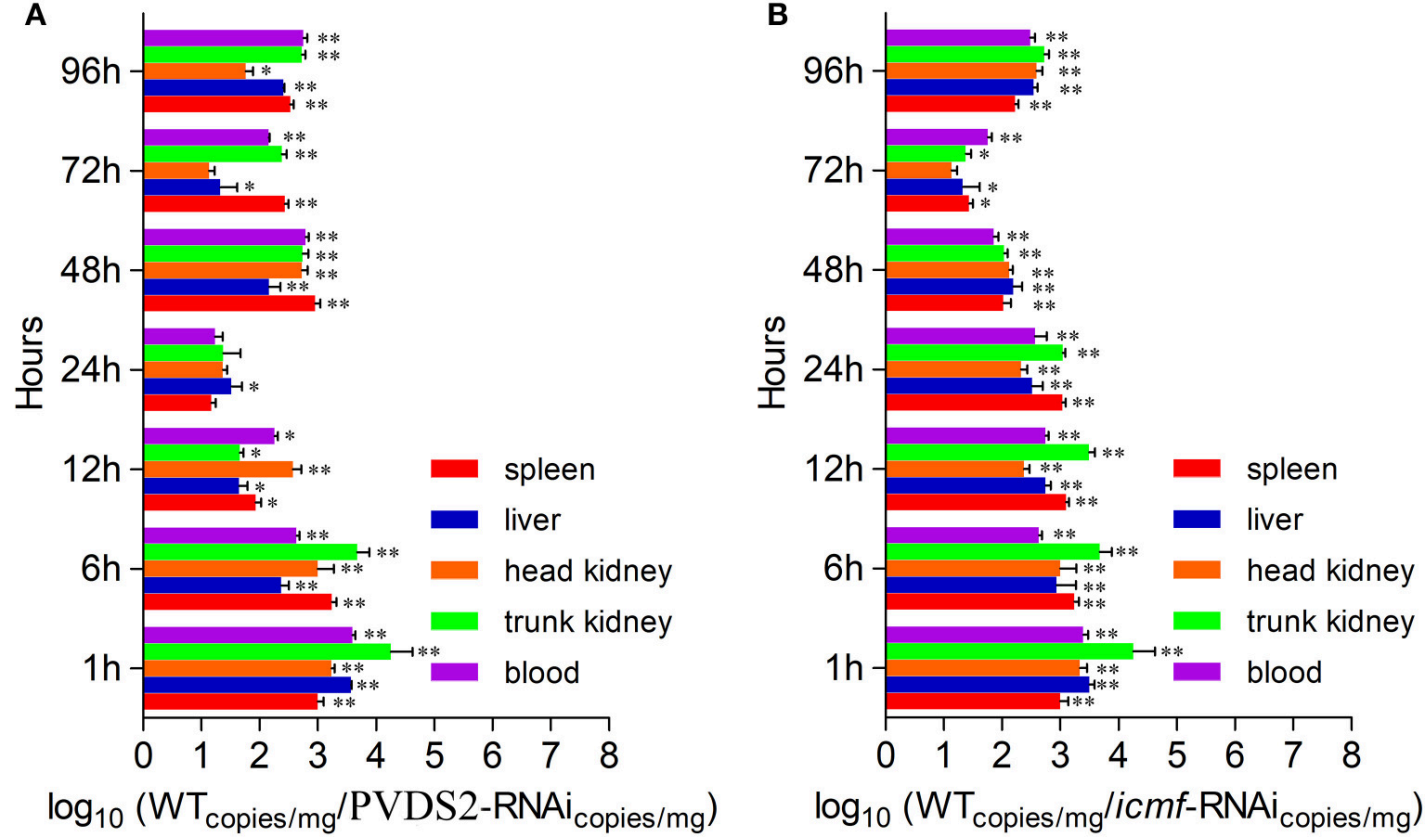
The dynamic distribution of RNAi strains in host. *E. coioides* were intrapleurally injected with WT *P. plecoglossicida* strain, PVDS2-RNAi strain and icmF-RNAi strain, respectively. The numbers of RNAi strains bacteria in spleen, liver, head kidney, trunk kidney and blood after injection of PVDS2-RNAi strain **(A)** and icmF-RNAi strain **(B)** were compared with the WT strain using qPCR at 1, 6, 12, 24, 48, 72, and 96 h. Data are shown as means ± SD from three independent biological replicates. * *P* < 0.05, **P < 0.01.

Since PVDS2 was the most sensitive to temperature change among these 5 genes, it may play a fundamental role in the *P. plecoglossicida* infection. To further validate this, the dynamic distribution of the PVDS2-RNAi strain in *E. coioides* was detected. The abundances of PVDS2-RNAi strain in the spleen, liver, head kidney, trunk kidney, and blood were significantly reduced compared with the wild-type *P. plecoglossicida* at most of the time points after injection (Figure 6A).

### Temperature-dependent regulation of the T6SS involved in virulence of *P. plecoglossicida*

**Figure 7:**
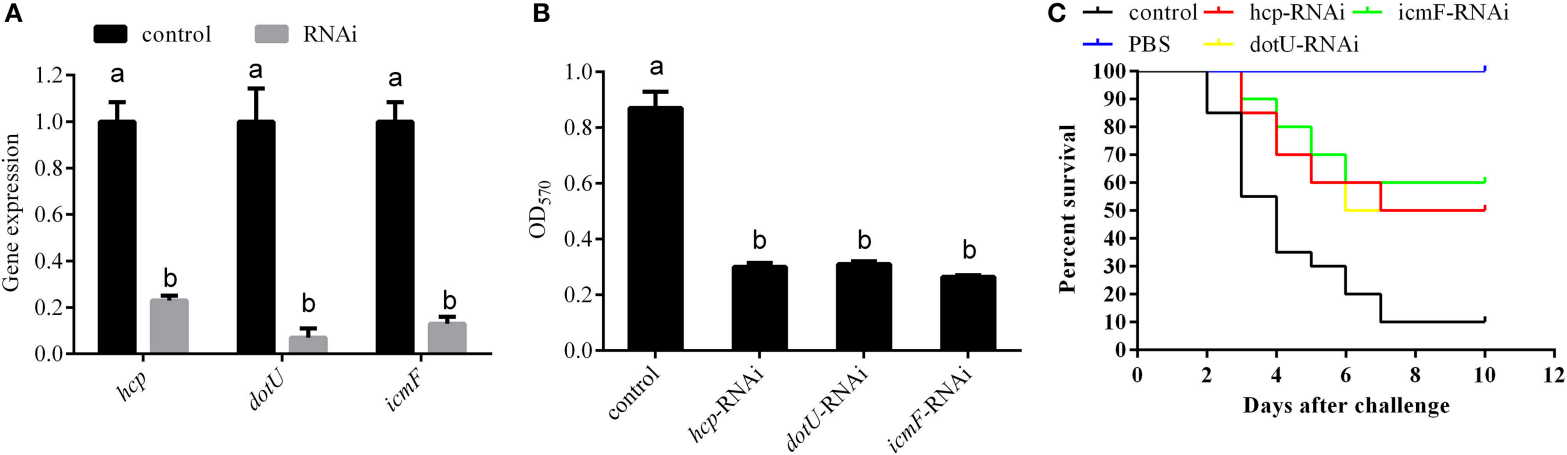
Temperature-dependent regulation of T6SS involved in virulence of *Pseudomonas plecoglossicida*. **(A)** qRT-PCR analysis of the expression of *hcp, dotU*, and *icmF* after stable gene silencing compared with the control. Data are presented as mean ± S.D. (*n* = 3). Means of treatments not sharing a common letter are significantly different at *P* < 0.05. **(B)** Biofilm formation of wild-type and stable silenced strains. Data are presented as mean ± S.D. (*n* = 6). Means of treatments not sharing a common letter are significantly different at *P* < 0.05. **(C)** The cumulative survival of *E. coioides* injected with wild-type and *hcp*-, *dotU*-, and *icmF*-RNAi strains during 10 days post-challenge.

According to the integrated analysis of the transcriptome and proteome, *hcp, dotU*, and *icmF*, which belong to T6SSs, also seem to be closely related to the temperature-dependent virulence of *P. plecoglossicida*. To validate this hypothesis, *hcp, dotU*, and *icmF* were silenced with RNAi, which significantly reduced their expression by 4.35-, 14.29-, and 7.69-fold, respectively (Figure 7A). Meanwhile, the biofilm formation (Figure 7B) and virulence (Figure 7C) of NZBD9 was significantly decreased in the silenced strains.

Since *icmF* was the most sensitive to temperature change among these 3 genes, it may play a fundamental role in the *P. plecoglossicida* infection. To further validate this, the dynamic distribution of the *icmF*-RNAi strain in *E. coioides* was detected. The abundances of *icmF*-RNAi strain in the spleen, liver, head kidney, trunk kidney, and blood were significantly reduced compared with the wild-type *P. plecoglossicida* at most of the time points after injection (Figure 6B).

## Discussion

Bacterial pathogenicity is complicated, calling for the expression of numerous virulence factors as well as other genes necessary for infection. These genes modulate the multiple steps of bacterial disease, including transmission, adhesion, penetration, survival, and host injury. Our results of RNA-seq showed that temperature had a significant influence on gene expression in NZBD9, indicating that the bacteria would focus their energy on different patterns in response to different growth temperatures, which may be the key to pathogenicity. Furthermore, considering the number of DEGs (Figures 1C,D), the distance between the PCA plots (Figure 1A) and that the samples cultured at 18°C had the tendency to cluster with samples cultured at 12°C rather than samples cultured at 28°C (Figure 1B), we hypothesized that the differences between samples cultured at 18 and 28°C were much greater than those between the samples cultured at 18 and 12°C. This provided additional evidence that the outbreaks of the white nodules disease were closely related to virulence regulation in *P. plecoglossicida*, instead of the traditional perspective that the disease could be caused by different types of pathogens and was only affected by the nutrient levels and immune function of the host.

There is a discrepancy between transcriptome analysis and proteome analysis. This is not unexpected since the proteome analysis was carried out on the extracellular and intracellular proteins separately. Furthermore, the secretion systems (such as T6SS, T3SS, T2SS and so on) were affected by temperatures in different ways, which might lead to the stranding or secretion of proteins. Taken together, the discrepancy between mRNA and the extracellular protein expression levels, or between mRNA and the intracellular protein expression levels is understandable. However, there may be other factors restricting concordant changes. For example, our use of transcriptome analysis and proteome analysis is a likely limitation that does not allow us to take into account differences in mRNA vs. protein half-lives. Another possible explanation was that post-transcriptional regulation may be one of the significant features for temperature-dependent pathogenicity of NZBD9. Importantly, despite the recognized limitations we outline, the concordance in the mRNA and protein changes in some virulence genes are still well. For example, the results of transcriptomic and proteomic analyses showed that genes involved in PVD synthesis and T6SS are specifically upregulated at the virulent temperature of 18°C, which is concurrent with the phenotypic changes. Silencing of the PVD-synthesis-related genes significantly reduces iron acquisition, growth, biofilm formation, distribution, and virulence. Silencing of the T6SS genes also leads to a notable reduction of biofilm formation, distribution and virulence.

Efficient iron uptaken by the bacteria is an essential factor for colonization. In the host, iron is either chelated by extracellular proteins strongly or present in the heme molecule of hemoproteins. *Pseudomonas* can acquire iron through the production of extracellular Fe^3+^-chelating molecules which was named as siderophores (including PVD and pyochelin). Compared to pyochelin, PVD has stronger affinity, greater abundance and a more complicated structure. Therefore, PVD is the chief siderophore of *Pseudomonas* (Cézard et al., 2015). PVD is a composite siderophore consisting of three parts: a chromophore common to all strains, a strain-specific peptide and a side-chain bound to the chromophore. The conserved chromophore of the molecule provides the catecholate group, which participates in the binding of Fe^3+^, while the peptide chain, which contains between 6 and 12 amino acids, is highly variable among different *Pseudomonas* species or even within a species. Interestingly, the high variability of the peptide chain among species is mainly due to the highly variable sidechain peptide synthetase (Cézard et al., 2015).

PVDs are the connection between pathogenicity and iron metabolism, and therefore, PVDs have aroused the interest of many scientists worldwide over the years. PVD has been proved to act as a signal molecule. For example, it can induce the production of the protease PrpL and the potent toxin exotoxin A (Lamont et al., 2002; Rédly and Poole, 2003, 2005; Visca et al., 2007; Cornelis, 2010). Likewise, PVD has been observed to participate in the establishment of biofilms (Banin et al., 2005; Patriquin et al., 2008; Glick et al., 2010).

T6SSs are widely distributed among gram-negative bacteria and are characterized by a needlelike appendage which is capable of delivering various toxins into both eukaryotic and bacterial cells. Among different species, T6SSs differ in their capacities to release a variety of effectors, which are commonly associated with two conserved proteins of the estimated 13 core T6SS components: hemolysin coregulated protein (Hcp), which forms the ejectable inner sheath of the nanotubular device; and valine-glycine repeat G protein (VgrG). The secretion of Hcp and VgrG is a hallmark of all T6SSs. ClpV and IcmF are two conserved components which have ATPase activity and act as energizers for T6SSs. IcmF is a membrane-localized protein belongs to T6SSs, which usually interacts with multiple T6SS components, for instance, DotU. In general, IcmF powers the secretion machinery assembly (Sexton et al., 2004; Zheng and Leung, 2007).

T6SS has been revealed to play a wide variety of physiological roles recently, for instance, roles in interbacterial interactions, host-symbiont communication, acute/chronic infection, and biofilm formation (Cascales, 2008; Filloux et al., 2008; Pukatzki et al., 2009; Weber et al., 2009; Hood et al., 2010; Schwarz et al., 2010; Ma et al., 2012). Interestingly, Weber et al. (2009) observed that a T6SS is essential for *Vibrio anguillarum* stress response and cell survival after exposed to various environmental stresses. Nevertheless, most studies of T6SSs are focused on the roles in cell-cell interactions and pathogenicity, while understanding about their roles in environmental stress responses is very limit.

Taken together, our results reveal that temperature regulates multiple virulence mechanisms in *P. plecoglossicida*, especially through iron uptake and toxin secretion.

The present study integrates transcriptomic and proteomic data, leading to new insights into the temperature-related pathogenesis of *P. plecoglossicida*, which would not have been easy to catch at either the protein or mRNA differential analyses alone, thus illustrating the power of multi-omics analyses in microbiology.

## Author contributions

Eight authors contributed to the article. QY and YS conceived the experiments. LH, YS, WL, QJ and YZ conducted the experiments. All authors assisted in the collection and interpretation of data. QY and LH wrote the manuscript.

### Conflict of interest statement

The authors declare that the research was conducted in the absence of any commercial or financial relationships that could be construed as a potential conflict of interest.

## Supplementary material

Figure S1 Histogram presentation of clusters of orthologous groups (COGs) classification of DEGs in 12°C group.

Figure S2 Histogram presentation of clusters of orthologous groups (COGs) classification of DEGs in 28°C group.

Table S1 Oligonucelotides used in producing shRNA for stable gene scilencing.

Table S2 Primers for qRT-PCR.

Table S3 The fold change of the main genes and proteins considered in this study.
