# Supplementary figures and images for: Integration of Transcriptomic and Proteomic Approaches Reveals the Temperature-Dependent Virulence of Pseudomonas plecoglossicida
Source: Front Cell Infect Microbiol. 2018 Jun 21;8:207. doi: 10.3389/fcimb.2018.00207 (PMC6021524; doi:10.3389/fcimb.2018.00207)

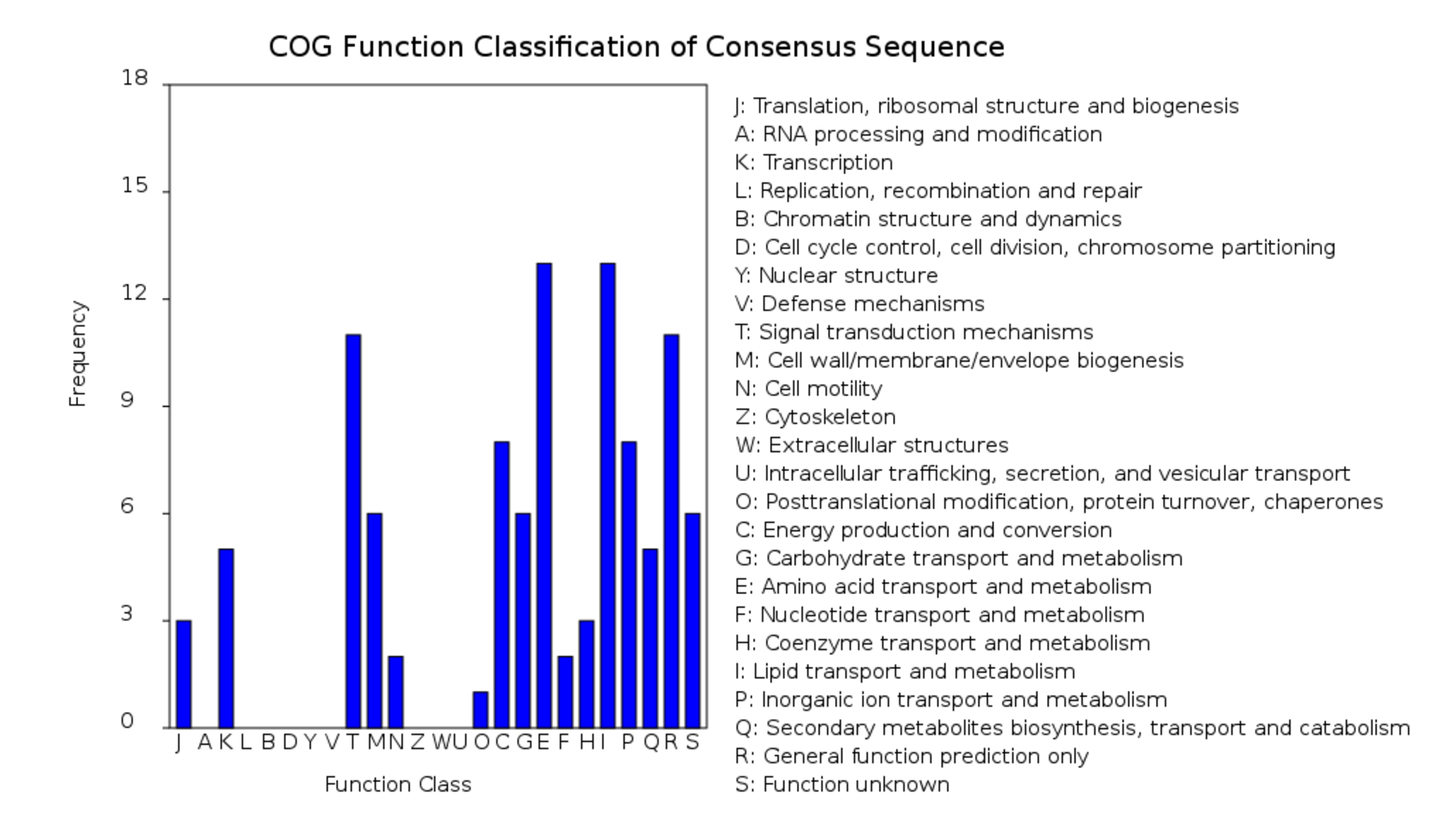

Supplement: Figure S1 — Histogram presentation of clusters of orthologous groups (COGs) classification of DEGs in 12°C group. [file Image_1.PNG]

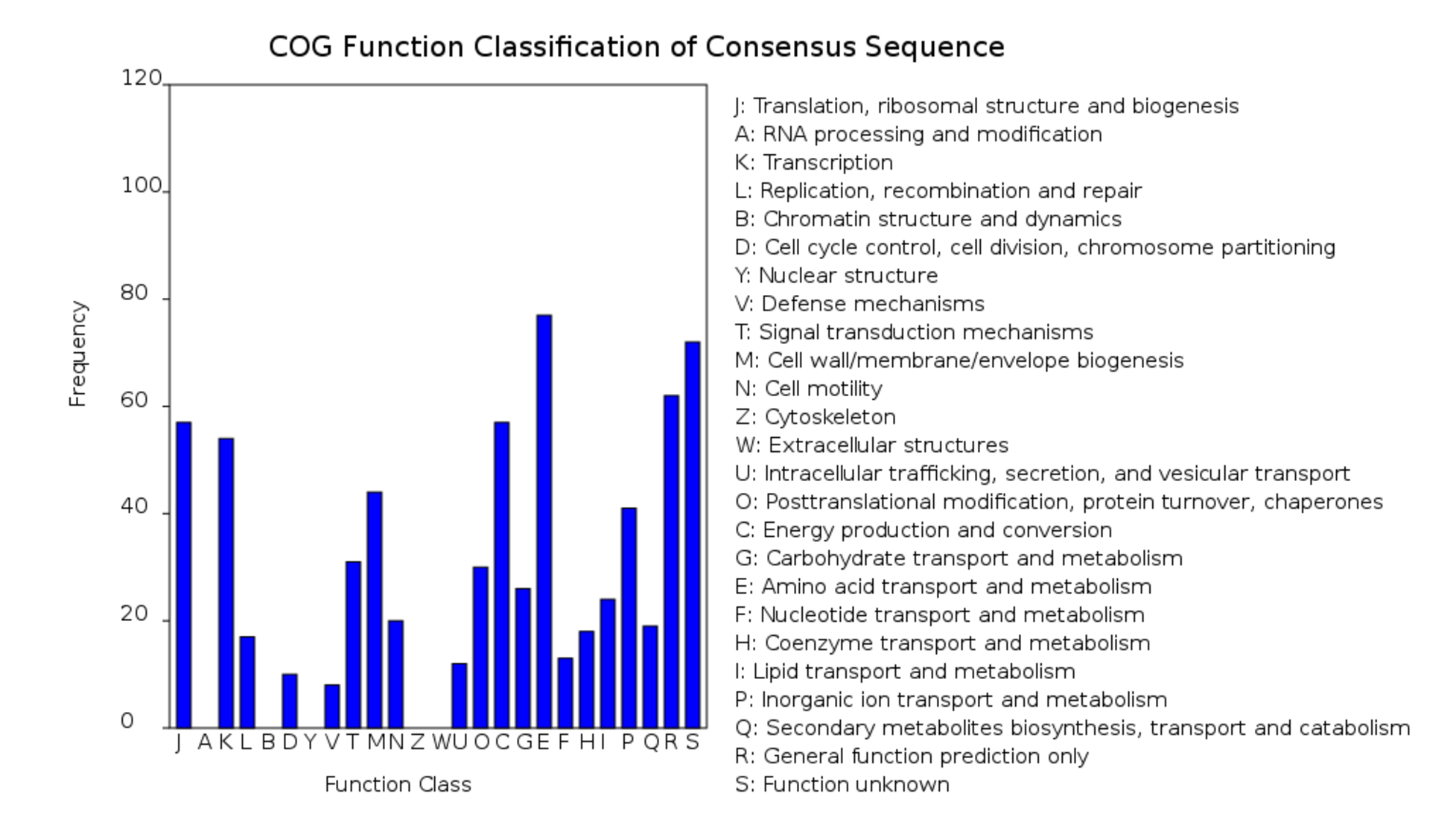

Supplement: Figure S2 — Histogram presentation of clusters of orthologous groups (COGs) classification of DEGs in 28°C group. [file Image_2.PNG]
