# Supplementary material for: Integration of Transcriptomic and Proteomic Approaches Reveals the Temperature-Dependent Virulence of Pseudomonas plecoglossicida
Source: Front Cell Infect Microbiol. 2018 Jun 21;8:207. doi: 10.3389/fcimb.2018.00207 (PMC6021524; doi:10.3389/fcimb.2018.00207)
Supplement: Table S1 — Oligonucelotides used in producing shRNA for stable gene scilencing. [file Table_1.DOC]

**Table S1** Oligonucleotides used in producing shRNA for stable gene silencing

| **Target gene** | **shRNA sequence for** **stable gene silence** |
| --- | --- |
| *pvds1* | F:5'-GATCCTGATCTCTTCGAGCAGCATTCAAGAGATGCTGCTCGAAGAGATCATTTTTTGCATG-3'  R:5'-CAAAAAATGATCTCTTCGAGCAGCATCTCTTGAATGCTGCTCGAAGAGATCAG -3' |
| *pvds2* | F:5'-GATCCTACCACGAGCGCCCAGGCTTCAAGAGAGCCTGGGCGCTCGTGGTATTTTTTGCATG-3'  R:5'-CAAAAAATACCACGAGCGCCCAGGCTCTCTTGAAGCCTGGGCGCTCGTGGTAG-3' |
| *pvds3* | F:5'-GATCCGTGACCTGTTCATGCATCTTCAAGAGAGATGCATGAACAGGTCACTTTTTTGCATG-3'  R:5'-CAAAAAAGTGACCTGTTCATGCATCTCTCTTGAAGATGCATGAACAGGTCACG-3' |
| *pvds4* | F:5'-GATCCATGACTGTCGCGGACCGTTTCAAGAGAACGGTCCGCGACAGTCATTTTTTTGCATG-3'  R:5'-CAAAAAAATGACTGTCGCGGACCGTTCTCTTGAAACGGTCCGCGACAGTCATG-3' |
| *pvds5* | F:5'-GATCCATGCAACTGCCTGCCCATTTCAAGAGAATGGGCAGGCAGTTGCATTTTTTTGCATG-3'  R:5'-CAAAAAAATGCAACTGCCTGCCCATTCTCTTGAAATGGGCAGGCAGTTGCATG-3' |
| *hcp* | F:5'-GATCCAAGTTTTTTGGTGTAAAAGACTTCAAGAGAGTCTTTTACACCAAAAAACTTTTTTTTGCATG-3'  R:5'-CAAAAAAAAGTTTTTTGGTGTAAAAGACTCTCTTGAAGTCTTTTACACCAAAAAACTTG-3' |
| *dotU* | F:5'-GATCCAATGTTCGCCGAATCGGTGGCTTCAAGAGAGCCACCGATTCGGCGAACATTTTTTTTGCAT-3'  R:5'-CAAAAAAAATGTTCGCCGAATCGGTGGCTCTCTTGAAGCCACCGATTCGGCGAACATTG-3' |
| *icmF* | F:5'-GATCCAGAAATTCTTCAACAGACGCATTCAAGAGATGCGTCTGTTGAAGAATTTCTTTTTTTGCATG-3'  R:5'-CAAAAAAAGAAATTCTTCAACAGACGCATCTCTTGAATGCGTCTGTTGAAGAATTTCTG-3' |
