# Supplementary material for: Integration of Transcriptomic and Proteomic Approaches Reveals the Temperature-Dependent Virulence of Pseudomonas plecoglossicida
Source: Front Cell Infect Microbiol. 2018 Jun 21;8:207. doi: 10.3389/fcimb.2018.00207 (PMC6021524; doi:10.3389/fcimb.2018.00207)
Supplement: Table S2 — Primers for qRT-PCR. [file Table_2.DOC]

**Table S2 Primers for qRT-PCR**

| **Gene** | **Primers** |
| --- | --- |
| *pvds1* | F:5'-TGATCTACACCTCCGGCTCC-3'  R:5'-GCGATGTCGAACGACAGTGA-3' |
| *pvds1* | F:5'-ATCGAACTGGGGGAAATCGA-3'  R:5'-AGGGTGCATTCCACATCGC-3' |
| *pvds1* | F:5'-TTGTCCGTGACCCGTCTGGA-3'  R:5'-CAGGCCGTCGATCACTACATCC-3' |
| *pvds1* | F:5'-AGCACAGTGCGCTGTACGATAT-3'  R:5'-CGGTCATAGCTGTAGCAGAAGC-3' |
| *pvds1* | F:5'-AGGTGCTCGCCGAGCTTCAA-3'  R:5'-AGGTGCCTGCTCCAGCGTTTC-3' |
| *hcp* | F:5'-GTCACCGTGGTCAAAGAGCTCA-3'  R:5'-TACATATGCCCTGACAACGCG-3' |
| *dotU* | F:5'-GATCACGACAACCGTGGCA-3'  R:5'-CGCGTAGAAGCTCTGGGAAA-3' |
| *icmF* | F:5'-GCAGATGCGTGCCTTCAATAC-3'  R:5'-CGACAATGACAGGCCACCA-3' |
| *gyrB* | F:5'-TGCTGAAGGACGAGCGTTCG-3'  R:5'-ATCATCTTGCCGACAACAGC-3' |
| *16S RNA* | F:5'-GTTGGGAGGAAGGGCAGTAAG-3'  R:5'-ATCTAGGCATTTCACCGCTACA-3' |
