# Supplementary material for: Integration of Transcriptomic and Proteomic Approaches Reveals the Temperature-Dependent Virulence of Pseudomonas plecoglossicida
Source: Front Cell Infect Microbiol. 2018 Jun 21;8:207. doi: 10.3389/fcimb.2018.00207 (PMC6021524; doi:10.3389/fcimb.2018.00207)
Supplement: Table S3 — The fold change of the main genes and proteins considered in this study. [file Table_3.DOCX]

**The fold change of the main genes and proteins considered in this study**

| 18 °C VS 12 °C | | | | | | |
| --- | --- | --- | --- | --- | --- | --- |
| mRNA | | | Intracellular proteins | | Extracellular proteins | |
| Gene | **Fold_change** | **p_value** | **Fold_change** | **p_value** | **Fold_change** | **p_value** |
| PVDS1 | 4.06 | 0.00005 | 1.77 | 0.00100 | ---- | ---- |
| PVDS2 | 3.03 | 0.00015 | 2.00 | 0.00005 | 1.17 | 0.00005 |
| dotU | 2.29 | 0.00005 | -1.12 | 0.01800 | 1.22 | 0.01500 |
| wbjB | 2.13 | 0.00050 | -1.92 | 0.01000 | -1.25 | 0.05700 |
| L321_05085 | 2.25 | 0.00005 | 1.10 | 0.42500 | -1.19 | 0.10800 |
| bcsA | 2.27 | 0.00013 | -1.17 | 0.01600 | 1.89 | 0.24300 |
| bcsB | 2.95 | 0.00100 | 1.18 | 0.37900 | 1.33 | 0.06300 |
| bcsC | 2.31 | 0.00005 | -1.24 | 0.00200 | ---- | ---- |
| tbdr | 2.23 | 0.00005 | -2.31 | 0.00005 | 1.33 | 0.00100 |
| PVDA | 2.26 | 0.00015 | ---- | ---- | -1.71 | 0.00005 |
| L321_07968 | 3.77 | 0.00005 | -1.98 | 0.00500 | -1.42 | 0.01500 |
| PVDS3 | 3.95 | 0.00005 | 1.56 | 0.00015 | ---- | ---- |
| PVDS4 | 3.03 | 0.00005 | 2.08 | 0.00013 | ---- | ---- |
| PVDS5 | 2.27 | 0.00059 | 2.13 | 0.00080 | -1.41 | 0.02300 |
| icmF | 3.03 | 0.00015 | 1.38 | 0.04300 | -2.24 | 0.00300 |
| rpmJ | 2.37 | 0.00005 | -3.96 | 0.00005 | -1.05 | 0.31700 |
| hcp | 2.14 | 0.00100 | -1.92 | 0.04000 | 4.81 | 0.00030 |
| copZ | 3.19 | 0.00005 | 1.35 | 0.10500 | 1.04 | 0.55600 |
| lysE | 5.95 | 0.00005 | ---- | ---- | ---- | ---- |
| mvaT | 2.05 | 0.00005 | -1.54 | 0.01100 | -1.36 | 0.02200 |
| L321_00372 | -2.04 | 0.00005 | ---- | ---- | ---- | ---- |
| rpoE | -2.15 | 0.00021 | ---- | ---- | ---- | ---- |
| hpaH | -2.11 | 0.00043 | ---- | ---- | ---- | ---- |
| metI | -2.18 | 0.00017 | ---- | ---- | ---- | ---- |
| fliN | -2.05 | 0.00005 | 1.15 | 0.16400 | -1.68 | 0.00100 |
| L321_15811 | -2.35 | 0.00005 | ---- | ---- | ---- | ---- |
| pycB | -4.18 | 0.00005 | 1.71 | 0.00100 | 1.18 | 0.01700 |
| L321_18107 | -2.66 | 0.00005 | ---- | ---- | ---- | ---- |
| fdhA | -2.30 | 0.00005 | 1.12 | 0.16100 | 1.22 | 0.00005 |
| wrbA | -2.49 | 0.00042 | 1.37 | 0.02600 | ---- | ---- |
| L321_20437 | -2.54 | 0.00005 | ---- | ---- | ---- | ---- |
| L321_20717 | -2.41 | 0.00016 | ---- | ---- | ---- | ---- |
| 18 °C VS 28 °C | | | | | | |
| mRNA | | | Intracellular proteins | | Extracellular proteins | |
| Gene | **Fold_change** | **p_value** | **Fold_change** | **p_value** | **Fold_change** | **p_value** |
| PVDS1 | 2.35 | 0.00033 | 2.54 | 0.00005 | ---- | ---- |
| PVDS2 | 3.21 | 0.00005 | 1.93 | 0.00005 | 1.62 | 0.00005 |
| dotU | 2.92 | 0.00011 | 1.37 | 0.00600 | 1.75 | 0.00005 |
| wbjB | 3.43 | 0.00005 | -1.12 | 0.12100 | 1.53 | 0.00005 |
| L321_05085 | 2.87 | 0.00005 | 1.03 | 0.82700 | 1.17 | 0.02600 |
| bcsA | 2.67 | 0.00035 | -1.71 | 0.00005 | 1.47 | 0.36700 |
| bcsB | 2.77 | 0.00014 | -1.28 | 0.23500 | -1.02 | 0.93000 |
| bcsC | 2.63 | 0.00005 | 1.19 | 0.00005 | ---- | ---- |
| tbdr | 3.04 | 0.00005 | -1.03 | 0.79300 | 1.21 | 0.01200 |
| PVDA | 2.20 | 0.00034 | ---- | ---- | 1.86 | 0.00100 |
| L321_07968 | 2.08 | 0.00005 | 1.03 | 0.83300 | 1.70 | 0.00500 |
| PVDS3 | 2.22 | 0.00005 | 1.51 | 0.00005 | ---- | ---- |
| PVDS4 | 2.44 | 0.00075 | 2.15 | 0.00100 | ---- | ---- |
| PVDS5 | 2.38 | 0.00018 | 1.87 | 0.00100 | 1.64 | 0.00100 |
| icmF | 3.96 | 0.00005 | -1.12 | 0.27400 | -1.23 | 0.02400 |
| rpmJ | 2.05 | 0.00005 | -2.04 | 0.00200 | 1.23 | 0.00400 |
| hcp | 2.39 | 0.00045 | 2.15 | 0.00200 | 6.41 | 0.00020 |
| copZ | 2.16 | 0.00005 | 1.26 | 0.08200 | 1.06 | 0.32900 |
| lysE | 17.92 | 0.00005 | ---- | ---- | ---- | ---- |
| mvaT | 4.62 | 0.00005 | 1.30 | 0.01900 | 1.28 | 0.00600 |
| L321_00372 | -2.03 | 0.00005 | ---- | ---- | ---- | ---- |
| rpoE | -2.18 | 0.00033 | ---- | ---- | ---- | ---- |
| hpaH | -2.72 | 0.00010 | ---- | ---- | ---- | ---- |
| metI | -2.15 | 0.00019 | ---- | ---- | ---- | ---- |
| fliN | -4.65 | 0.00005 | 1.20 | 0.13200 | -1.09 | 0.57700 |
| L321_15811 | -3.52 | 0.00005 | ---- | ---- | ---- | ---- |
| pycB | -2.87 | 0.00005 | -1.26 | 0.00700 | 1.64 | 0.00005 |
| L321_18107 | -2.10 | 0.00020 | ---- | ---- | ---- | ---- |
| fdhA | -2.21 | 0.00070 | -2.20 | 0.00005 | -227.95 | 0.00005 |
| wrbA | -2.26 | 0.00042 | -2.01 | 0.00005 | ---- | ---- |
| L321_20437 | -2.76 | 0.00005 | ---- | ---- | ---- | ---- |
| L321_20717 | -2.29 | 0.00018 | ---- | ---- | ---- | ---- |
